# Teachers’ Emotional Commitment: The Emotional Bond That Sustains Teaching

**DOI:** 10.3390/jintelligence13120158

**Published:** 2025-12-02

**Authors:** Olena Kostiv, Antonio F. Rodríguez-Hernández, Jonathan Delgado Hernández

**Affiliations:** Department of Developmental and Educational Psychology, Faculty of Psychology, University of La Laguna, 38200 San Cristóbal de La Laguna, Spain; antrodri@ull.edu.es (A.F.R.-H.); extjdelgado@ull.edu.es (J.D.H.)

**Keywords:** teacher emotional commitment, empathy, teacher–student relationship, affectivity, social–emotional skills, psychometrics

## Abstract

This study introduces and validates the construct of Teacher Emotional Commitment (CED), understood as the conative–behavioral dimension that characterizes the emotional bond that teachers establish with their students. To this end, two complementary studies were conducted in the Autonomous Community of the Canary Islands (Spain), with the aim of: to empirically isolate the factorial structure of CED and differentiating it from related constructs, such as empathy; to analyze its presence in both active teachers and those in initial training; and to test the theoretical model’s validity by expanding the sample and enlarging the response scale. Study 1 involved 854 practicing teachers and 701 teachers in training, following a validation process that included exploratory and confirmatory factor analysis, as well as item response theory models. The results showed a four-factor structure: loving proactivity, teacher compassion, instructional commitment, and communicative affectivity, with adequate reliability and discriminant validity indices with respect to empathy. Study 2, with an expanded sample of 2096 participants, confirmed the robustness of the model. The findings allow us to consider CED as a psychological competence that can be trained, with relevant implications for improving the educational relationship, student learning, and the emotional well-being of teachers.

## 1. Introduction

From a psychoeducational perspective, when focusing on the personal elements of the instructional setting, learning depends on teaching; in other words, a student’s learning will be conditioned by the support provided by their teachers to make that “educational miracle” possible. This perspective situates teachers as central protagonists in the educational process and reinforces the idea that high-quality education emerges from the work of good teachers.

Although a vast body of research has examined teacher effectiveness in instructional tasks, the present study emphasizes a fundamental aspect: the emotional bond that sustains teaching. The aim is to isolate the “emotional heart” of this relationship, expressed through attitudes and interaction patterns designed to strengthen the teacher–student alliance. Emotional connection with students has been consistently identified as a key predictor of the quality of educational relationships, regardless of the analytical perspective adopted (Bayram Özdemir and Özdemir 2020; Pianta 1999; Roorda et al. 2019; Yu et al. 2018). We refer to this conative–behavioral construct as Teacher Emotional Commitment (CED, acronym in Spanish).

Studying CED is relevant for several reasons: to determine whether it constitutes a psychological construct that characterizes how teachers bond with their students, how it manifests in practice, and to what extent it differs from related concepts such as empathy. Additionally, it is crucial to examine whether CED appears exclusively among practicing teachers or also emerges during preservice teacher training, thereby situating it within the developmental trajectory of professional growth. Offering empirical evidence on these questions is especially important, as school learning requires an intentional willingness to learn, which is only facilitated when teachers are perceived as trustworthy figures.

Empirically isolating this emotional connection not only enables the analysis of its influence on the teaching–learning process but also supports its transfer through preservice and in-service training programs. Moreover, CED has implications for teachers’ affective stability and mental health, since previous research (Rodríguez 2025) has demonstrated that it operates as a moderating variable in teachers’ vulnerability to emotional strain in the teaching profession.

To conceptually define the construct, we present an overview of the main theoretical models and areas of psychology to identify similarities and differences with related constructs and offer a theoretical synthesis that allows us to define CED.

Conceptually, CED can be framed within multiple psychological models. The ecological systems model (Bronfenbrenner 1976, 1977a, 1977b) situates it within the microsystem, as it focuses on the dyadic teacher–student relationship. Attachment theory (Bowlby 1969; Ainsworth 1991) provides evidence of the relevance of emotional bonds in human development, which underlie the teacher–student connection, particularly in early childhood, where teachers often serve as secondary attachment figures (Ainsworth 1991; Díaz et al. 2004). Previous studies have shown that attachment patterns influence the quality of teacher–student relationships across educational stages (Bergin and Bergin 2009; Howes and Ritchie 1999; McCartney et al. 2004; Sierra and Moya 2012). This allows us to understand the effect that CED would have when students establish an emotional connection with the teacher. Similarly, social support theory (Tardy 1985) contributes to conceptualizing CED, especially in terms of emotional support, understood as perceptions of trust, care, and empathy that students develop through their interaction with teachers (Liu et al. 2016; Romano et al. 2020; Strati et al. 2017; Suldo et al. 2009).

Although there are theoretical parallels between emotional support and CED, there are clearly differentiating aspects. On the one hand, the perspective from which the link is described. In the case of emotional support, it is the students who describe their perception, so it would be a hetero-evaluative perspective; whereas, in the case of CED, it is the teacher themselves who refers, in a self-evaluative way, to the way they relate to their students. Another difference is that, while the emotional support dimension is a generic category used to capture the way in which the teacher interacts with their students, the CED refers to specific interaction patterns through which the emotional bond is manifested.

Going into detail about the dimensions that have been analyzed in the teacher–student relationship, the literature has placed special emphasis on the component of closeness (Pianta 2001), also referred to as satisfaction (Ang 2005; Ang et al. 2020), support (Hughes et al. 2008), or warmth (Wu and Hughes 2015). This dimension is characterized by attitudes of warmth, support, affection, and respect, through which teachers provide emotional support to students. These attributes refer to a meaningful emotional connection, considered a core element of teacher–student interaction (Longobardi et al. 2016; Sabol and Pianta 2012; Toste et al. 2015). It should also be noted that CED should not be confused with the dimension of emotional engagement assessed by the Engaged Teacher Scale (Klassen et al. 2013), which focuses on the degree to which teachers identify with their professional role. Although there may be a certain relationship with the dimension of social commitment to students, CED is specifically oriented toward the affective quality of the teacher–student relationship, defined by the willingness of teachers to maintain an emotional bond that favors the educational process.

From the perspective of clinical psychology, CED can be compared to the therapeutic alliance, understood as the positive relationship established between therapist and client, a key factor in both adult and child psychotherapy (Norcross and Lambert 2018), regardless of the specific intervention or moderating variables (Carvalho et al. 2015; Wampold 2015; Murphy and Hutton 2017). CED is conceptualized as a bond with educational functionality, whose purpose is to help learning. This defining characteristic, associated with the teaching function, is crucial, since CED acquires meaning not only from its emotional content but also from its orientation toward the goals of education.

A key issue concerns the needs of students addressed by this bond and the motivations of teachers who sustain it. To understand this conative–motivational dimension of CED, self-determination theory (Deci and Ryan 1985) offers a relevant framework, highlighting the human need for relatedness and its influence on students’ personal, social, and academic adjustment. Within this framework, the concept of teacher involvement is particularly relevant, as it directly connects with our construct.

Another defining feature of CED is its nature as an emotional competence, understood as a socio-affective “know-how” that teachers can develop through training. This perspective aligns with proposals emphasizing the need to teach teachers how to construct positive relationships with students (Gallagher et al. 2019). In this regard, CED can be situated within the category of emotional-bonding competencies in the Competent Emocreativity Model (Rodríguez Hernández et al. 2025). Such competencies enable effective interaction beyond personal experience, involving empathy, affective communication, and the establishment of affective bonds. While the empirical isolation of CED was achieved through teacher self-report, positioning it as a general and potential psychological characteristic, its conative–attitudinal dimension supports its conceptualization as a trainable capacity, similar to intrapersonal emotional competencies (Bisquerra Alzina 2005; Bueno García et al. 2005; Fernández-Berrocal and Ruiz-Aranda 2008; Teruel-Melero 2000).

It is also necessary to theoretically distinguish CED from empathy to establish conceptual divergent validity before testing it empirically. While both constructs fall within the category of emotional-bonding competencies (Rodríguez Hernández et al. 2025), suggesting potential correlations, important differences exist. Dispositional and affective empathy implies sharing emotions or “feeling with” another person (Fernández-Pinto et al. 2008; Sánchez-Queija et al. 2006; Singer and Lamm 2009; Chiu and Yeh 2018; Håkansson-Eklund and Summer-Meranius 2021). CED, in contrast, is expressed through concern, affection, and the motivation to help, resembling compassion. Thus, empathy is best understood as a cognitive–affective process, while CED represents a conative–motivational process. Moreover, empathy facilitates the creation of positive relationships (Huang et al. 2020; Makoelle 2019).

In light of these considerations, CED is defined as a form of interaction situated in the microsystem of the dyadic teacher–student relationship. It is grounded in teachers’ self-perceptions of how they manifest their emotional bond with students and is characterized by warmth, care, affection, and respect, which provide affective support. Unlike affective empathy, Teacher Emotional Commitment possesses a conative–motivational quality oriented toward sustaining an affective alliance that serves an educational purpose. Furthermore, CED can be understood as a socio-affective competence of teachers, which can be systematically trained and developed through teacher education.

The aims of this study are threefold: (a) to validate the psychoeducational construct of Teacher Emotional Commitment by empirically isolating its factorial structure and differentiating it from empathy; (b) to verify the presence of CED among teachers in training, to explore its developmental trajectory in professional growth across educational stages; and (c) to test the theoretical model of CED by expanding the sample and enlarging the response scale. The research was organized into two complementary studies: Study 1, addressing the first two objectives, and Study 2, addressing the third.

The guiding research questions were

1. Does Teacher Emotional Commitment exist as a psychological construct that characterizes the teacher–student relationship?
2. How is this affective bond manifested in teaching practices?
3. Is CED different from other constructs, such as empathy, that are also present in teacher–student interactions?
4. Does CED emerge as a characteristic of in-service teachers only, or is it also present among preservice teachers, thereby situating the construct within the developmental trajectory of teacher professional growth?

**Study 1**

## 2. Materials and Methods

### 2.1. Participants

The total sample consisted of 1555 participants, divided into two groups: active teachers (*n* = 854) and trainee teachers (*n* = 701), all from the Autonomous Community of the Canary Islands (Spain), participating in different phases of the study.

Active teachers: A total of 854 teachers from early childhood, primary, and secondary education participated, belonging to public, charter, and private schools, with the first two being the most represented. The sample was distributed in three phases: 98 teachers participated in the initial construction of the construct, 85 in the exploratory phase, and 671 in the validation phase. The average age in the final phase was 41.7 years (SD = 10.74), with 71.6% women and 28.4% men. The average teaching experience was 14.7 years (SD = 10.41), ranging from less than one year to 41 years. In terms of professional performance, 39.6% were tutors, 27.3% were tutors and specialists, 25.3% were specialists, and the rest were support teachers or linked to specific programs. In the Spanish educational system, different teaching roles coexist within schools. The tutor acts as the main classroom teacher, responsible for the academic, personal, and social monitoring of students, as well as for communication with families and coordination with the teaching staff. Specialist teachers possess specific training in particular areas, such as music, physical education, foreign languages, or special needs education, and teach only their specialization while collaborating with tutors in curricular planning. Support teachers provide targeted intervention for students with specific educational needs or who participate in reinforcement and inclusion programs, either within or outside the regular classroom setting. Tutors, specialists, and support teachers share educational responsibility for the same students, and their collaboration ensures the integration of general and specific approaches to educational attention.

Trainee teachers: A total of 701 university students participated: 167 in their third year of the Bachelor’s Degree in Early Childhood Education, 294 in their third year of the Bachelor’s Degree in Primary Education, and 240 in the Master’s Degree in Teacher Training. Their ages ranged from 19 to 53 (M = 23.6; SD = 5.00), with 68.2% women and 31.8% men. In terms of degree programs, 75.8% were from Social Sciences and Law, 12.7% from Arts and Humanities, 5.2% from Engineering and Architecture, 3.8% from Sciences, and 2.5% from Health Sciences. The vast majority (95.2%) indicated that they had not received previous training in emotional education.

### 2.2. Procedure

The validation process of the CED construct followed the methodological guidelines of Muñiz and Fonseca-Pedrero (2019) and was developed in eight successive phases:

1. Initial exploratory phase: an initial questionnaire with three open-ended questions (see Table 1) was administered online to 98 in-service teachers, with the goal of identifying expressions linked to CED and capturing the qualitative complexity of the construct.

2. Content analysis: responses were categorized by three expert judges, who generated conceptual categories for each question, providing a first approximation of possible dimensions of the construct.

3. Validation of categories: internal consistency of the categories was assessed by five additional judges (psychologists and teachers across educational levels). Each judge was provided with the grouped responses, the provisional categories, and their definitions, and was asked to classify each response into the proposed categories. Fleiss’ kappa (κ) was calculated using Epidat 4.2.

4. Construction of the item bank: between six and nine items were created for each conceptual category. From this initial bank, exploratory studies were conducted to refine the instrument and add new items derived from theoretical review and expert judgment. Refinement criteria included: (a) exclusion of items referring to the reasons behind teachers’ feelings, to isolate the construct; (b) elimination of items forming a single factor; (c) factor loadings below 0.30; (d) elimination of items without detriment to internal consistency or explained variance; and (e) elimination of ambiguous items. The preliminary questionnaire consisted of 19 items.

5. Pilot study: the questionnaire was administered to 85 primary school teachers. Exploratory factor analysis (EFA) and internal consistency analysis were conducted, including confidence intervals for reliability coefficients.

6. Validation study: once the factorial structure was established, the questionnaire was administered to 671 teachers. Following item refinement and model fit analyses, the questionnaire was reduced from 19 to 14 items. The instrument was distributed digitally (via online form sent to schools) and in paper format (administered by undergraduate and master’s students). In all cases, participants were informed about the study objectives and the confidentiality of data.

7. Divergent validation: joint administration of the Interpersonal Reactivity Index (IRI) together with the Teacher Emotional Commitment Questionnaire to 671 teachers, comparing the dimensions of the CED with those of the IRI.

8. Validation in trainee teachers: the questionnaire was administered to 701 students in training, in both online and paper formats. Implementation was supported by university faculty responsible for the courses in which the questionnaire was applied. Participants were informed about the study objectives, data confidentiality and provided informed consent. The reference variables included teaching experience. Participants with one year or more of teaching experience were excluded to ensure homogeneity and equivalence in terms of professional practice.

### 2.3. Instruments

Interpersonal Reactivity Index (IRI; Davis 1980, 1983). This is a widely used self-report measure for assessing empathy from a multidimensional perspective that integrates cognitive and emotional components. The instrument consists of 28 items distributed across four dimensions of seven items each: Perspective Taking, Fantasy, Empathic Concern, and Personal Distress. The response format is Likert scale with five options (1 = ‘does not describe me well’; 5 = ‘describes me very well’). In terms of the nature of the dimensions, Perspective Taking and Fantasy assess cognitive processes: the first refers to the tendency to adopt the point of view of others, while the second assesses the imaginative ability to identify with fictional characters in literature or film. Empathic Concern and Personal Distress, on the other hand, reflect emotional processes: the first captures feelings of compassion, affection, and concern for the distress of others (other-oriented), while the second assesses responses of anxiety and distress when observing the negative experiences of others (self-oriented). The psychometric properties of the Spanish version (Pérez-Albéniz et al. 2003) are comparable to those of the original version, with Cronbach’s alpha coefficients ranging from 0.69 to 0.80. A particular feature of the adaptation is the location of item 13, which in the Spanish version is included in the Empathetic Concern dimension, rather than in Personal Distress.

Teacher Emotional Commitment Questionnaire (CUCODE). Developed for this study, based on the theoretical conceptualization of CED. Items were hypothesized to cluster into four factors: Loving Proactivity, Communicative Affectivity, Teacher Compassion, and Instructional Commitment. The items have been translated to facilitate understanding, but it is essential to adapt them to the specific context of the place where they will be used.

### 2.4. Statistical Analyses

The validation process included several analyses. First, inter-rater reliability was estimated using Fleiss’s Kappa coefficient (κ) to assess the degree of agreement among raters in the classification of conceptual categories. Subsequently, EFA was conducted using principal component extraction and varimax rotation after verifying normality assumptions through skewness and kurtosis indices. Internal consistency was evaluated using Cronbach’s alpha and McDonald’s omega. Item Response Theory (IRT) models were applied to estimate item fit and discrimination capacity, allowing further refinement of the questionnaire and subsequent EFA on the reduced version. Confirmatory Factor Analysis (CFA) was then performed with robust weighted least squares estimation (WLSMV), including teaching experience as a covariate. Divergent validity was analyzed through Spearman correlations between CED and IRI dimensions. Finally, CFA was conducted with the trainee teacher sample to assess model fit, and EFA was used to compare item distribution across both populations. Analyses were conducted using SPSS v25 (IBM Corp. 2017), R (R Core Team 2024), and ULLRToolbox.

## 3. Results

### 3.1. Content Validity 

Interrater agreement among the five judges yielded a Fleiss’ kappa coefficient of κ = 0.71 (*p* < .001), which, according to Altman’s (1991) criteria, indicates a good level of agreement.

### 3.2. Exploratory Factor Analysis

Descriptive statistics of the 19 preliminary items revealed means ranging from M = 2.85 (item 13) to M = 3.59 (item 9), suggesting a tendency toward high scores. The values for skewness (−0.82 to 0.21) and kurtosis (−1.43 to 1.15) did not exceed the absolute values of 3 and 10, respectively (Kline 2016), suggesting an adequate fit to normality. Standard deviations ranged from 0.49 to 1.00.

The Kaiser–Meyer–Olkin (KMO) measure of sampling adequacy was 0.846, and Bartlett’s test of sphericity was significant (*p* < .000), supporting the suitability of the data for factor analysis. Following Kaiser–Gutman’s criterion, a four-factor solution was obtained, with eigenvalues greater than 1, explaining 61.24% of the total variance. Specifically, the first component explained 22%, the second 13.99%, the third 12.93%, and the fourth 12.30% of the variance. Table 2 presents the rotated component matrix, showing the factorial structure of TEC. Items with loadings below 0.30 were suppressed for clarity.

The examination of the content of the items, together with the theoretical review carried out, made it possible to establish a structure composed of four clearly differentiated factors. Factor 1: Loving proactivity refers to the emotional availability of teachers, expressed in receptive behaviors and verbal and non-verbal expressions of affection towards students. This factor was made up of six items (1, 4, 9, 10, 13 and 15), which include statements such as the importance of showing affection in the classroom or the willingness to offer comfort and affection. Factor 2: Teacher compassion reflects the ability of teachers to accept and forgive their students, regardless of their characteristics or any situations of conflict that may arise. It consisted of five items (2, 5, 7, 16 and 17), focusing on aspects such as patience, careful treatment, attention to emotional state and the ability to forgive. Factor 3: Instructional commitment describes attitudes and actions aimed at promoting the teaching–learning process, such as the search for new methodologies, the systematic monitoring of students, or the effort to ensure meaningful learning. This factor consisted of four items (3, 6, 14, and 18). Finally, Factor 4: Communicative Affectivity, groups together the verbal and non-verbal expressions of an affective nature that teachers use when addressing their students, such as the use of words of gratitude, the transmission of happiness, or friendly looks. This factor consisted of four items (8, 11, 12, and 19).

### 3.3. Internal Consistency

Reliability analyses evidenced adequate internal consistency for the CUCODE and its dimensions in the 19-item version. Table 3 shows Cronbach’s alpha coefficients for both the overall scale and each of the factors. For the complete questionnaire (19 items), a coefficient of α = 0.897 (95% CI = 0.861–0.927) was obtained. At the factor level, the values ranged from 0.724 to 0.862. In addition, Table 4 reports the corrected item–total correlations and the alpha coefficient if an item were deleted. All corrected item-total correlations were above the reference value of 0.30, which supports the relevance of each item on the scale. Furthermore, in no case did the removal of an item produce an increase in the overall alpha value, confirming the adequacy of the 19-item structure and the internal stability of the instrument.

The results obtained allow us to move towards a more detailed analysis of the items through the TRI in order to examine the extent to which each item contributes to the measurement of the CED construct.

### 3.4. Item Response Theory 

Response frequencies were examined to ensure that no categories had zero values. This procedure led to the elimination of item 2 (“*Soy paciente con mis alumnos y alumnas, me tomo las cosas con calma, sin desesperarme, ni alterarme*”) and item 5 (“*Trato a mi alumnado con delicadeza y afecto, por ejemplo, cuando me dirijo a ellos/as no alzo la voz, ni utilizo gestos violentos*”), since no participant in the sample (*N* = 671) selected response category 1.

The analysis, based on the unrestricted discrimination model, yielded the following discrimination coefficients for each factor of the CUCODE:

5. Loving Proactivity: parameters ranged from 1.40 to 3.82. The most discriminating item was Item 4 (3.82), followed by Item 1 (2.73) and Item 13 (2.71), while Items 9 and 10 showed the lowest values.
6. Teacher Compassion: values ranged from 1.12 to 2.73. Item 17 was the most discriminating (2.73), while Item 7 showed the lowest value.
7. Instructional Commitment: parameters varied between 1.09 and 2.16. Item 18 obtained the highest value (2.16), while Item 14 was the least discriminating.
8. Affective Communication: values ranged between 1.64 and 2.84. Item 19 was the most discriminating (2.84) and Item 12 the least.

ANOVA comparisons between the restricted and unrestricted discrimination models were significant for all factors, confirming a better fit for the unrestricted model. Specifically, results yielded *p* < .001 for Loving Proactivity, Teacher Compassion, and Affective Communication, and *p* = .006 for Instructional Commitment.

In line with the principle of parsimony, items 9, 10, and 14 were removed due to low discrimination values, both within their factor and across the scale as a whole. Although Item 7 displayed similar behavior, it was retained to ensure that the corresponding factor contained at least three items. Finally, the item characteristic curves indicated a higher probability that teachers would respond in categories 3 and 4, compared to categories 1 and 2, in addition to a general tendency toward mid-to-low levels in the assessed trait.

### 3.5. Exploratory Factor Analysis

After eliminating three items via IRT and two items due to the absence of responses in the lowest response category, an EFA was conducted again to confirm the four-factor structure of CED with a 14-item scale. Results of the Kaiser–Meyer–Olkin measure of sampling adequacy (0.90) and Bartlett’s test of sphericity (χ^2^ = 3045.222; *p* < .001) confirmed the suitability of the data for factor analysis (see Table 5). A varimax rotation was applied with four fixed factors to extract. To facilitate interpretation of the components, the same criteria adopted in the previous EFA were maintained.

The elimination of items 2, 5, 9, 10, and 14 did not compromise the four-factor factorial structure previously defined for the construct under study. Item 16 has a higher factor loading in the Communicative Affectivity dimension. However, it was decided to keep it in the Teacher Compassion dimension, considering its conceptual relevance and the fact that it continues to have considerable weight in the corresponding factor.

### 3.6. Confirmatory Factor Analysis 

Descriptive statistics for the items were first computed. Results indicated values close to zero for skewness (−1.26 to −0.24) and kurtosis (−1.13 to 1.37), suggesting an approximately normal distribution (Kline 2016). Standard deviations ranged from 0.53 to 1.03.

**Figure 1:**
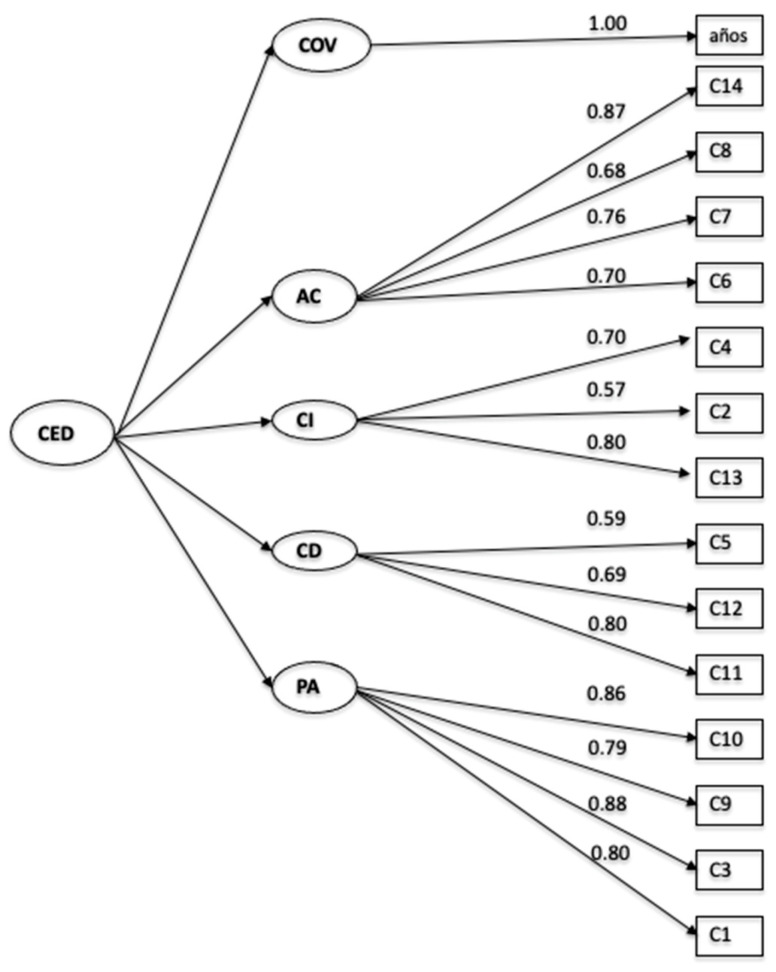
AFC Model of Teacher Emotional Commitment.

The four-factor model, including the variable “years of teaching experience,” was evaluated through CFA using robust weighted least squares estimation (WLSMV), given the ordinal nature of the scale. The model yielded χ^2^(81) = 220.710, *p* = .000, an expected outcome for samples larger than 200 and robust fit indices. Model fit indices were χ^2^/df = 2.72; NFI = 0.986; CFI = 0.991; RMSEA = 0.052 (90% CI: 0.044–0.060); and SRMR = 0.053, all indicating a satisfactory fit. All factor loadings were significant, ranging from 0.57 (good) to 0.88 (excellent), surpassing the recommended minimum threshold of 0.40 (Comrey and Lee 1992) (see Figure 1).

### 3.7. Internal Consistency 

Cronbach’s alpha and McDonald’s omega coefficients for the factors of the CUCODE, after confirmation of the four-factor structure via CFA, are presented in Table 6.

### 3.8. Divergent Validity

Correlations were observed between the dimensions of both constructs with a small effect size (Table 7), according to Cohen’s criteria (Cohen 1988). More specifically, Perspective Taking showed a small effect with Loving Proactivity and Instructional Commitment, and a medium effect with Teacher Compassion and Affective Communication. The fantasy dimension has only a small effect on Affective Communication, without significant correlations with the other CED dimensions. Empathic Concern exhibited small effects across all CED dimensions. By contrast, Personal Distress demonstrated small and inverse correlations with all CED variables.

### 3.9. Confirmatory Factor Analysis 

**Figure 2:**
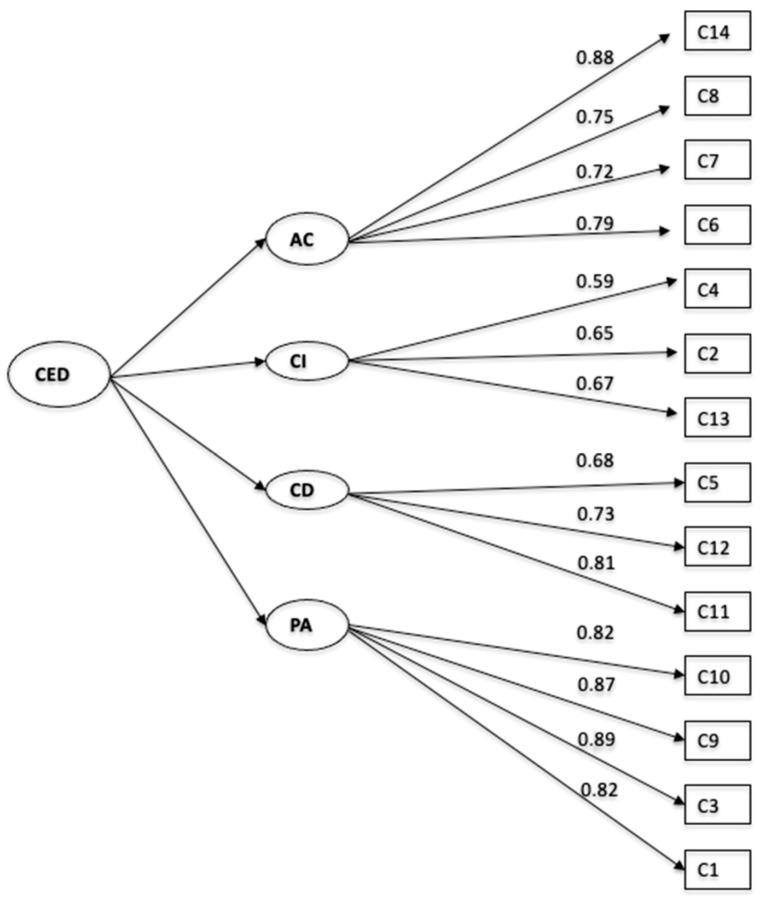
CFA of Teacher Emotional Commitment in trainee teachers.

This was applied to the total sample of trainee teachers, using weighted least squares maximum likelihood estimation (WLSMV). The goodness-of-fit indices indicated a good fit for the theoretical model: χ^2^(71) = 141.075, *p* < .000; χ^2^/gl = 1.98; NFI = 0.994; CFI = 0.997; RMSEA = 0.038 (90% CI: 0.029, 0.047); SRMR = 0.040. All factor loadings were significant, with values between 0.59 (good) and 0.89 (excellent), above the recommended minimum of 0.40 (Comrey and Lee 1992), see Figure 2.

McDonald’s Omega and Cronbach’s alpha internal consistency coefficients yielded the following values for each dimension of Teacher Emotional Commitment: loving proactivity ω = 0.78, α = 0.91; teaching compassion ω = 0.69, α = 0.78; instructional commitment ω = 0.56, α = 0.67; and communicative affectivity ω = 0.79 and α = 0.87.

### 3.10. Exploratory Factor Analysis 

Descriptive statistics indicated skewness values (−0.97 to −0.02) and kurtosis values (−1.24 to 0.48) close to zero, suggesting an approximately normal distribution (Kline 2016). The Kaiser–Meyer–Olkin measure of sampling adequacy (0.93) and Bartlett’s test of sphericity (χ^2^ = 3887.641; *p* < .000) confirmed the suitability of the data for conducting an EFA.

Principal components extraction with varimax rotation was applied, fixing four factors according to the dimensions comprising the questionnaire. To facilitate interpretation of the components, the same criteria adopted in previous EFAs were used. The factorial solution explained 64.64% of the total variance: Component 1 = 29.31%; Component 2 = 15.27%; Component 3 = 10.20%; Component 4 = 9.87%.

To compare item distribution across factors in the two populations, Table 8 presents the rotated component matrix for active teachers alongside the corresponding matrix for trainee teachers.

As shown in Table 8, Components 1 and 2, corresponding to Loving Proactivity and Teacher Compassion, grouped the same items as in the original model. However, the dimension Affective Communication did not emerge as a unique factor, as its items were distributed across Components 1 and 2. Thus, the dimension Instructional Commitment was divided between Components 3 and 4.

Regarding the structure coefficients of the dimensions for the trainee teacher sample compared to the original sample, the following patterns were observed: in Loving Proactivity, factor loadings were higher for Items 3, 9, and 10 and lower for Item 1; in Teacher Compassion, Item 5 displayed a lower loading, whereas Items 11 and 12 also showed reduced values; in Instructional Commitment, loadings were higher than in the original factor; and finally, in Affective Communication, loadings were lower for Items 7 and 8 but higher for Items 6 and 14. Furthermore, as in the original structure, Items 6, 10, 11, 12, and 14 shared high cross-loadings (greater than 0.30) with other questionnaire dimensions, which is theoretically coherent.

In general terms, the questionnaire retained an adequate fit to the four-factor model in the trainee teacher sample. Nevertheless, results also suggested the presence of an alternative three-factor structure. To test this emerging hypothesis, a second exploratory factor analysis was conducted, fixing three factors as the solution. Sampling adequacy indices confirmed the viability of this analysis (KMO = 0.93; Bartlett χ^2^ = 3887.641; *p* < .000). The rotated factorial solution explained 59.13% of the total variance, distributed as follows: Component 1 = 27.50%; Component 2 = 20.04%; Component 3 = 11.63%. The rotated component matrix for this solution is presented in Table 9.

It can be observed that the items corresponding to the Instructional Commitment dimension are distributed in a single factor. Similarly, the Loving Proactivity and Teacher Compassion dimensions contain the same items as the initial model. However, as in the previous exploratory factor analysis, the Communicative Affectivity dimension is not configured as an independent factor, but rather its items are distributed between the Loving Proactivity and Teacher Compassion components. Possible explanations for this result are discussed below.

**Study 2**

## 4. Materials and Methods

### 4.1. Participants

The total sample included 2096 participants. The group of active teachers consisted of 786 people, and the group of trainee teachers included 1310 participants, considered for the corresponding analyses. The mean age of the total sample was 24.3 years (SD = 18). In terms of gender, 579 participants identified as male, 1482 as female, 9 as ‘other,’ and 26 did not respond.

### 4.2. Procedure

The questionnaire was administered in digital format, using an online form. Participants were informed in advance about the objectives of the research, the voluntary nature of their participation, and the confidentiality of the data. To optimize the quality of the measurement, the response scale was expanded from four to eight points, and the order of the items was randomized.

### 4.3. Instruments

Interpersonal Reactivity Index (IRI; Davis 1980, 1983). Described in Study 1.

Teacher Emotional Commitment Questionnaire (Cuestionario de Compromiso Emocional Docente, CUCODE, Kostiv and Rodríguez-Hernández 2022). This is a self-report measure consisting of 14 items grouped into four dimensions, designed to assess teachers’ perceptions of their ability to establish an emotional bond with their students (Appendix A). The items cover different forms of interaction between teachers and their students, which are expressed in a series of verbal and non-verbal manifestations. The response format corresponds to an eight-point Likert scale (1 = I do not identify with this at all, 8 = I identify with this very much). The dimensions that compose the questionnaire are as follows: Loving Proactivity (four items) refers to the teacher’s emotional availability when interacting with their students, that is, being receptive and affectionate through verbal and non-verbal expressions. Communicative Affectivity (four items) covers the verbal and non-verbal expressions of affection that teachers use when addressing their students. Teacher Compassion (three items) refers to the ability of teachers to accept and forgive their students regardless of their characteristics or the events in which they may have been involved. Instructional Commitment (three items) describes the attitudes and actions that teachers take to promote the teaching–learning process. The psychometric properties are included in the development of this study. The items have been translated to facilitate understanding, but it is essential to adapt them to the specific context of the place where they will be used.

Trait Meta-Mood Scale (TMMS-24; Fernández-Berrocal et al. 2004; original version by Salovey et al. 1995). This is a self-report scale that assesses individual differences in people’s ability to pay attention to their emotions, discriminate between them, and repair them. The scale consists of three dimensions, each with eight items: Emotional Awareness (ability to perceive and express feelings appropriately), Emotional Clarity (understanding emotional states), and Emotional Repair (ability to regulate emotional states correctly). The response scale ranges from 1 to 5, where 1 means ‘strongly disagree’ and 5 means ‘strongly agree.’ The psychometric properties of the Spanish version (Fernández-Berrocal et al. 2004) indicate Cronbach’s alpha coefficients ranging from 0.60 to 0.83.

Emotional Communication Questionnaire (CCE, acronym in Spanish; Hernández-Jorge et al. 2022). This is a self-report instrument designed to assess emotional communication in educational and healthcare contexts. It consists of 14 items with a five-point Likert scale (1 = never, 5 = always). The items are distributed across three dimensions: Communicative Proactivity (6 items), characterized by a positive attitude towards initiating and maintaining communication; Openness and Authenticity (5 items), showing oneself to others spontaneously and without deception; and Listening (3 items), strategies that ensure affective and emotional communication. It has adequate internal consistency in each of its components.

### 4.4. Statistical Analysis

All statistical analyses were conducted using R (version 4.4.0; R Core Team 2024) through the RStudio interface (version 2024.12.0.467; Posit Team 2024). The number of factors to retain was determined with the *nfactors* package. CFA was performed using *lavaan*, with the WLSMV estimator applied, given the ordinal nature of the items. Finally, IRT models were estimated with the *mirt* package.

Fit criteria were defined a priori: χ^2^/df ≤ 3 (satisfactory), CFI and TLI ≥ 0.90 (acceptable) and ≥ 0.95 (excellent), RMSEA ≤ 0.08 (adequate) and ≤ 0.05 (excellent), and SRMR ≤ 0.08 (acceptance threshold). The decision on the number of factors was guided by the Bayesian Information Criterion (BIC) provided by *nfactors*. For internal consistency, both Cronbach’s alpha and McDonald’s omega were computed.

## 5. Results

### 5.1. Confirmatory Factor Analysis 

This was applied to the sample of active teachers (*n* = 786; data cleaning, *n* = 770) to verify the degree of fit of the established model. The fit indices for the four-factor model were as follows: χ^2^/df = 25.41, CFI = 0.96, TLI = 0.95; RMSEA = 0.12 (90% CI: 0.11–0.12); SRMR = 0.03.

### 5.2. Confirmatory Factor Analysis

This was applied to the sample of trainee teachers (*n* = 1310; data cleaning, *n* = 1002) to confirm the invariance of the model. The fit indices obtained were as follows: χ^2^/df = 12.68, CFI = 0.999, TLI = 0.998; RMSEA = 0.108 (90% CI: 0.102–0.114); SRMR = 0.048. Although the CFI, TLI, and SRMR values indicate a good fit, the RMSEA index suggests a poor fit of the four-factor model in this group.

### 5.3. Internal Consistency

Cronbach’s alpha and McDonald’s omega coefficients for the instrument and its factors in the total sample of participants are presented in Table 10. The results confirm the internal reliability of the instrument overall and by dimension.

### 5.4. Item Response Theory 

The analysis performed on the total sample indicated adequate psychometric performance of the 14 items (see Table 11). All items had discrimination parameters (a1) greater than 1.0, with a range observed between 1.292 (item 14) and 6.826 (item 5) and a mean of 4.95 (SD = 1.60), indicating moderate to very high discrimination, confirming their usefulness in differentiating between participants with different levels of the latent trait.

The difficulty parameters (d1 to d7) show a consistent ordering pattern (d1 > d2 > ... > d7) across all items, validating the logical progression of the categories. However, many items have a high difficulty threshold (>3), indicating that the scale is more accurate at medium-high levels of the trait and may be less sensitive at low levels.

### 5.5. Divergent Analysis 

Pearson correlations were calculated between the CUCODE factors and the IRI, CCE, and TMMS-24 dimensions in the total sample of participants, see Table 12. The results show low correlations except for the moderate association between Empathic Concern on the IRI (Factor 3) and the four CUCODE factors (r = 0.45–0.50), which points to a certain conceptual proximity that will be addressed in the discussion.

## 6. Discussion

The purpose of this study was to empirically validate the psychoeducational construct Teacher Emotional Commitment, to delineate its factorial structure and differentiate it from related constructs such as empathy, and to analyze its presence among trainee teachers to explore its developmental nature within professional growth. In addition, we sought to test the robustness of the theoretical model by expanding both the sample size and the response range of the scale, thereby ensuring stronger psychometric evidence.

The results of the first study, based on exploratory and confirmatory factor analyses, confirmed the existence of CED as a psychoeducational construct characterizing the teacher–student relationship. Its structure is organized into four dimensions: Loving Proactivity, Teacher Compassion, Instructional Commitment, and Affective Communication, represented by a total of 14 items. Together, these dimensions describe the emotional bond established by teachers with their students through verbal and non-verbal expressions of affection, care, and concern. Item response theory analyses allowed refinement of the questionnaire by eliminating items with lower discriminative power while retaining those best able to differentiate among participants. It also offers the advantage of providing invariant parameters with respect to the sample, ensuring that item discriminative power remains stable across contexts (Matas 2001). Regarding internal consistency, analyses indicated adequate reliability levels at both the global and dimensional levels. Comparisons between Cronbach’s alpha and McDonald’s omega revealed slight differences attributable to the limited range of the initial four-point scale, which led to clustering of responses in the upper categories. As noted by Dunn et al. (2014), alpha can overestimate reliability when its assumptions are not met, whereas omega is more appropriate for ordinal scales with few categories (Frías-Navarro 2021). These findings answer the first research question and align with previous literature underscoring the relevance of teacher–student emotional connection as a determinant of relationship quality, regardless of perspective or analytic approach (Bayram Özdemir and Özdemir 2020; Pianta 1999; Roorda et al. 2019; Yu et al. 2018).

With respect to the second research question, the four identified factors illustrate how CED is manifested in teaching practice. Loving Proactivity reflects teachers’ affective availability, expressed through receptive behaviors and affective verbal and non-verbal expressions toward students. Teacher Compassion encompasses the capacity to accept and forgive students regardless of their personal characteristics or situations in which they are involved. Instructional Commitment refers to the attitudes and actions teachers implement to facilitate teaching–learning processes. Finally, Affective Communication focuses on affective verbal and non-verbal expressions deployed during everyday interaction with students.

These dimensions are consistent with the main teacher–student relationship dimensions described in the literature (Ang et al. 2020; Pianta 2001; Hughes et al. 2008; Wu and Hughes 2015). The dimension of closeness may be considered the overarching dimension encompassing Loving Proactivity, Teacher Compassion, and Affective Communication, as it is defined by warmth, affection, and open communication between teachers and students (Ang et al. 2020; Pianta 2001). However, our dimensions extend beyond this by providing concrete actions that operationalize the emotional bond developed by teachers with students, thereby enabling the identification of specific behavioral and verbal manifestations that enrich understanding of the construct. The literature also refers to another dimension, instrumental support (Ang 2005; Ang et al. 2020), which differs from the conceptualization of social support theory but is closely related to our factor of Teacher Compassion. Instrumental support is defined as the degree to which teachers maintain a supportive attitude and genuine concern for their students, offering help and support when needed. Analysis of the items suggests that both dimensions share unconditional concern and supportive attitudes toward students, always from the teacher’s perspective. Nevertheless, our conceptualization of Teacher Compassion introduces a distinctive nuance by situating this attitude within a broader framework of emotional commitment. This perspective allows us to understand that the teacher–student affective bond is not limited to closeness or instrumental support but integrates acceptance, forgiveness, and continuous accompaniment, thereby providing a more comprehensive conceptualization of CED.

Regarding the third research question, the divergent validity analyses demonstrated that the dimensions comprising our construct capture content distinct from empathy dimensions. Although small correlations were observed between both constructs, this is theoretically consistent, since empathy falls within the same category of emotional linkage competencies as CED (Rodríguez Hernández et al. 2025). Moreover, empathy is widely considered a trait and a crucial ability in teachers, enabling the creation of positive relationships (Huang et al. 2020; Makoelle 2019) and, in our case, fostering teachers’ emotional commitment to their students. Importantly, while empathy is conceptualized as a cognitive–affective process, our construct is located in the conative–motivational sphere.

Analyses with the trainee teacher sample provided evidence addressing the fourth research question. Although future teachers acknowledged the importance of affective expressions and displayed a mental representation of CED similar to that of active teachers, they exhibited difficulties in clearly distinguishing the Affective Communication dimension. This suggests that they perceive it as implicit within the interactions represented by the other two dimensions. Such a finding implies that professional experience operates as a developmental moderator, gradually enabling the differentiation of this dimension as an independent factor. Consequently, CED appears to emerge already in initial teacher education, albeit with a less differentiated structure, which reinforces its developmental character within professional growth. This outcome is consistent with prior studies on emotional competencies in initial teacher education, which highlight that students recognize the importance of emotions in the classroom as central to holistic student development and express favorable attitudes toward their future application in teaching practice (Hernández-Amorós and Urrea-Solano 2017).

With respect to the second study, results confirmed and extended those of the first. First, instrument reliability and item discriminative capacity reached optimal levels, in some cases with extremely high internal consistency coefficients. This finding opens the possibility of exploring, in future research, a reduction in the number of items without compromising construct validity. Second, the construct validity of CED was reconfirmed through confirmatory analyses applied to both active and trainee teachers. Structural models demonstrated excellent fit indices, with the exception of RMSEA, whose less favorable values are understandable given the characteristics of the sample and the instrument. These results reinforce the stability of the theoretical model and its applicability across different teacher populations. Third, findings supported divergent validity, confirming that CED is distinct from empathy, emotional communication, and perceived emotional intelligence. Nevertheless, the observed correlations with empathy and emotional communication are theoretically coherent, as CED cannot be understood in isolation but rather within a broader framework of emotional linkage competencies shaping teaching practice. It is plausible that these competencies represent specific manifestations of a higher-order construct that organizes and structures how teachers establish and sustain affective bonds in educational contexts.

This approach is related to the theoretical bases used to support the construct. On the one hand, we delimited the emotional dimension of social support theory (Tardy 1985; Liu et al. 2016; Romano et al., 2020; Strati et al. 2017; Suldo et al. 2009), offering a more nuanced understanding from the teacher’s perspective and specifying the attitudes and behaviors that materialize such bonds in educational relationships. On the other hand, results confirmed that CED is related to competencies such as empathy and emotional communication while maintaining divergent validity. This supports the hypothesis that these competencies form a triadic set clustered around a meta-construct, referred to as Competent Educational Love (Kostiv and Rodríguez-Hernández 2022; Rodríguez Hernández and Espinosa 2024; Rodríguez 2025), whose further exploration will be the focus of future research. Moreover, CED was clearly differentiated from intrapersonal emotional competencies, such as those measured by the TMMS-24, while showing a specific association with the ability to connect with others’ feelings, as assessed through the Empathic Concern dimension of the IRI (Chiu and Yeh 2018; Singer and Lamm 2009; Håkansson-Eklund and Summer-Meranius 2021). This finding is consistent with the idea that to commit emotionally to another person, one must first connect with what that person feels.

Despite the stronger validation of the construct through dual empirical evidence, several issues remain for a deeper understanding. First, the construct has been validated from the teacher’s perspective; therefore, it is necessary to incorporate students’ perceptions to determine whether there is congruence between both viewpoints and, if not, to identify the variables mediating such discrepancies. Second, CED should be analyzed in diverse educational contexts, taking into account factors such as student heterogeneity and situational conflict in daily interactions. In this regard, it would also be highly relevant to explore how teachers, both pre-service and in-service, self-evaluate their emotional commitment depending on whether reciprocal interactions with students are perceived or not. Moreover, longitudinal research would be valuable to determine whether the perceptions of the CED held by the same participants remain stable from the initial training stage to professional practice. It would also be pertinent to investigate how CED interacts with other linkage competencies that may mediate the quality of teacher–student interaction, such as empathy, and their influence on outcomes relevant to teaching and learning effectiveness, including academic achievement, classroom climate, and students’ emotional competence development. Finally, future studies could analyze how this construct manifests across different cultural contexts, considering variations in socially acceptable expressions of affect across cultures.

In sum, although these scientific gaps remain, the studies presented here provide robust, empirically tested evidence of the existence of an emotional bond that sustains teaching, going beyond teacher empathy. Moreover, we hypothesize that acknowledging CED as a key factor in educational quality represents a step toward achieving a school that enables learning from what is truly “essential”.

## Figures and Tables

**Table 1:** Open-ended questions in the initial questionnaire.

Question	Statement
1	What is the predominant feeling you experience toward your students?
2	How does that feeling manifest itself?
3	Why do you feel that way?

**Table 2:** Rotated component matrix of the 19 preliminary items.

Item	Component			
	F1	F2	F3	F4
13. Suelo ser afectuoso/a con ellos/as a través de abrazos y/o besos (*I tend to be affectionate with them through kisses and/or hugs*).	0.872			
4. Expreso a mi alumnado el amor que siento hacia ellos/as (*I express my love for my students*).	0.828			
1. Las muestras de cariño son imprescindibles en mis clases (*Showing affection is essential in my classes*).	0.785			
15. Soy generoso en la relación con mi alumnado: doy afecto sin esperar que ellos/as me lo devuelvan (*I am generous in my relationship with my students: I give affection without expecting them to return it*).	0.760			
9. Cuando mis alumnos y alumnas lo necesitan, estoy disponible para que encuentren consuelo en mí (*When my students need it, I am available for them to find comfort in me*).	0.517			
10. Siento la necesidad de protegerlos y cuidarlos (*I feel the need to protect and care for them*).	0.505			
7. Aunque tenga roces y conflictos con mi alumnado, mi sentimiento de afecto hacia ellos/as no cambia (*Even if I have disagreements and conflicts with my students, my feelings of affection towards them do not change*).		0.743		
5. Trato a mi alumnado con delicadeza y afecto, por ejemplo, cuando me dirijo a ellos/as no alzo la voz, ni utilizo gestos violentos (*I treat my students with kindness and affection. For example, when I speak to them, I do not raise my voice or use violent gestures*).		0.728		
17. Soy capaz de perdonar a mi alumnado, aunque me haga daño (*I am able to forgive my students, even if they hurt me*).		0.637		
2. Soy paciente con mis alumnos y alumnas, me tomo las cosas con calma, sin desesperarme, ni alterarme (*I am patient with my students, I take things calmly, without getting desperate or upset*).		0.575		
16. Estoy pendiente de su estado de ánimo para ayudarlos (*I am attentive to their mood in order to help them*).		0.402		
3. Me preocupo en buscar y aplicar nuevas metodologías de enseñanza (*I make an effort to find and apply new teaching methodologies*).			0.779	
18. Soy exigente conmigo mismo/a para que mi alumnado aprenda lo mejor posible (*I am demanding of myself so that my students learn as well as possible*).			0.744	
14. Me esfuerzo para que mi alumnado aprenda de manera divertida (*I strive to make learning fun for my students*).			0.611	
6. Realizo un seguimiento de mi alumnado para evaluarlo mejor (*I monitor my students’ learning in order to evaluate them better*).			0.505	
19. Hablo con cariño cuando me relaciono con ellos/as (*I speak kindly when I interact with them*).				0.675
8. Mantengo miradas amables hacia ellos/as (*I maintain a friendly gaze towards them*).				0.674
11.Transmito alegría en la relación con mi alumnado, y esto queda reflejado en mis expresiones (*I convey joy in my relationship with my students, and this is reflected in my expressions*).				0.583
12. Cuando me dirijo hacia mi alumnado utilizo palabras de gratitud hacia ellos/as (*When I address my students, I use words of gratitude towards them*).				0.527
Eigenvalues	4.20	2.66	2.46	2.34
Cumulative variance explained	22	36	48.94	61.24

Note: F1 (Loving Proactivity), F2 (Teacher Compassion), F3 (Instructional Commitment), and F4 (Communicative Affectivity).

**Table 3:** Internal consistency (global and by factor) for the CUCODE, 19 item.

Components	Elements	α	IC (95%)
CUCODE	19	0.897	0.861–0.927
Loving proactivity	6	0.862	0.810–0.903
Teacher compassion	5	0.771	0.683–0.841
Instructional commitment	4	0.724	0.613–0.809
Communicative affectivity	4	0.771	0.679–0.842

**Table 4:** Reliability statistics for the 19 item.

Item	Corrected Item–Total	Cronbach’s Alpha If the Item Has Been Deleted
1	0.685	0.887
2	0.360	0.896
3	0.442	0.895
4	0.627	0.889
5	0.446	0.894
6	0.344	0.897
7	0.601	0.890
8	0.465	0.894
9	0.676	0.888
10	0.397	0.896
11	0.646	0.889
12	0.656	0.888
13	0.556	0.894
14	0.524	0.892
15	0.723	0.885
16	0.549	0.892
17	0.524	0.892
18	0.332	0.897
19	0.593	0.891

**Table 5:** Matrix of rotated components and total variance explained for the 14 items.

	Component			
	PA	AC	CD	CI
1. (1) Las muestras de cariño son imprescindibles en mis clases (*Showing affection is essential in my classes*).	0.836			
3. (4) Expreso a mi alumnado el amor que siento hacia ellos/as (*I express my love for my students*).	0.803			
9. (13) Suelo ser afectuoso/a con ellos/as a través de abrazos y/o besos (*I tend to be affectionate with them through kisses and/or hugs*).	0.788			
10. (15) Soy generoso en la relación con mi alumnado: doy afecto sin esperar que ellos/as me lo devuelvan (*I am generous in my relationship with my students: I give affection without expecting them to return it*).	0.527	0.344	0.447	
8. (12) Cuando me dirijo hacia mi alumnado utilizo palabras de gratitud hacia ellos/as (*When I address my students, I use words of gratitude towards them*).		0.752		
7. (11) Transmito alegría en la relación con mi alumnado, y esto queda reflejado en mis expresiones (*I convey joy in my relationship with my students, and this is reflected in my expressions*).		0.652		
14. (19) Hablo con cariño cuando me relaciono con ellos/as (*I speak kindly when I interact with them*).	0.413	0.623		
11. (16) Estoy pendiente de su estado de ánimo para ayudarlos (*I am attentive to their mood in order to help them*).		0.520	0.441	
6. (8) Mantengo miradas amables hacia ellos/as (*I maintain a friendly gaze towards them*).	0.388	0.391		
5. (7) Aunque tenga roces y conflictos con mi alumnado, mi sentimiento de afecto hacia ellos/as no cambia (*Even if I have disagreements and conflicts with my students, my feelings of affection towards them do not change*).			0.723	
12. (17) Soy capaz de perdonar a mi alumnado, aunque me haga daño (*I am able to forgive my students, even if they hurt me*).		0.389	0.708	
2. (3) Me preocupo en buscar y aplicar nuevas metodologías de enseñanza (*I make an effort to find and apply new teaching methodologies*).				0.805
4. (6) Realizo un seguimiento de mi alumnado para evaluarlo mejor (*I monitor my students’ learning in order to evaluate them better*).				0.658
13. (18) Soy exigente conmigo mismo/a para que mi alumnado aprenda lo mejor posible (*I am demanding of myself so that my students learn as well as possible*).		0.457		0.548
Eigenvalues	2.83	2.46	1.72	1.59
Variance %	20.22	17.63	12.30	11.41
Cumulative %	20.22	37.85	50.15	61.56

Note: PA (Loving Proactivity), AC (Communicative Affectivity), CD (Teaching Compassion) and CI (Instructional Commitment); (number) = initial item numbering.

**Table 6:** Internal consistency coefficients for each CUCODE factor, 14 items.

Components	Elements	α AFC	ω AFC
Loving proactivity	4	0.89	0.72
Teacher compassion	3	0.72	0.63
Instructional commitment	3	0.73	0.61
Communicative affectivity	4	0.84	0.75

**Table 7:** Spearman correlations between empathy dimensions and CED.

		TP	F	PE	MP	PA	CD	CI	AC
P	Correlation coefficient	1.000							
	Sig. (two-tailed)	.							
F	Correlation coefficient	0.214	1.000						
	Sig. (two-tailed)	0.000	.						
PE	Correlation coefficient	0.328	0.434	1.000					
	Sig. (two-tailed)	0.000	0.000	.					
MP	Correlation coefficient	−0.221	0.146	0.122	1.000				
	Sig. (two-tailed)	0.000	0.000	0.002	.				
PA	Correlation coefficient	0.241	0.061	0.196	−0.097	1.000			
	Sig. (two-tailed)	0.000	0.120	0.000	0.014	.			
CD	Correlation coefficient	0.312	0.060	0.129	−0.164	0.512	1.000		
	Sig. (two-tailed)	0.000	0.129	0.001	0.000	0.000	.		
CI	Correlation coefficient	0.293	0.024	0.133	−0.111	0.340	0.440	1.000	
	Sig. (two-tailed)	0.000	0.537	0.001	0.005	0.000	0.000	.	
AC	Correlation coefficient	0.346	0.079	0.206	−0.162	0.603	0.536	0.452	1.000
	Sig. (two-tailed)	0.000	0.045	0.000	0.000	0.000	0.000	0.000	.

Note: RI dimensions: TP (Perspective Taking), F (Fantasy), PE (Empathic Concern) and MP (Personal Distress); CUCUDE dimensions: PA (Loving Proactivity), AC (Communicative Affectivity), CD (Teaching Compassion) and CI (Instructional Commitment).

**Table 8:** Comparison of factor loadings across construct dimensions by group.

	Active Teachers					Trainee Teachers			
	PA	AC	CD	CI		PA/AC	CD/AC	CI	CI
Item 1	0.836				Item 9	0.844			
Item 3	0.803				Item 3	0.833			
Item 9	0.788				Item 1	0.806			
Item 10	0.527	0.344	0.447		Item 14	0.703	0.316		
Item 8		0.752			Item 10	0.690	0.326		
Item 7		0.652			Item 8	0.515	0.359		
Item 14	0.413	0.623			Item 11	0.502	0.303	0.419	
Item 11		0.520	0.441		Item 5		0.811		
Item 6	0.388	0.391			Item 6	0.449	0.602		
Item 5			0.723		Item 12	0.326	0.548	0.393	
Item 12		0.389	0.708		Item 7	0.404	0.440	0.303	
Item 2				0.805	Item 13			0.859	
Item 4				0.658	Item 4		0.307		0.810
Item 13		0.457		0.548	Item 2				0.744

Note: PA (Loving proactivity); AC (Communicative affectivity), CD (Teaching compassion) and CI (Instructional commitment); Underlined item corresponds to AC.

**Table 9:** Matrix of rotated components, with three fixed factors in trainee teachers.

Item	Component		
	PA/AC	CD/AC	CI
9. Suelo ser afectuoso/a con ellos/as a través de abrazos y/o besos (*I tend to be affectionate with them through kisses and/or hugs*).	0.825		
3. Expreso a mi alumnado el amor que siento hacia ellos/as (*I express my love for my students*).	0.825		
1. Las muestras de cariño son imprescindibles en mis clases (*Showing affection is essential in my classes*).	0.811		
10. Soy generoso/a en la relación con mi alumnado: doy afecto sin esperar que ellos/as me lo devuelvan (*I am generous in my relationship with my students: I give affection without expecting them to return it*).	0.677	0.363	
14. Hablo con cariño cuando me relaciono con ellos (*I speak kindly when I interact with them*).	0.660	0.453	
8. Cuando me dirijo hacia mi alumnado utilizo palabras de gratitud hacia ellos/as (*When I address my students, I use words of gratitude towards them*).	0.481	0.464	
12. Soy capaz de perdonar a mi alumnado, aunque me hagan daño (*I am able to forgive my students, even if they hurt me*).		0.693	
5. Aunque tenga roces y conflictos con mi alumnado, mi sentimiento de afecto hacia ellos/as no cambia (*Even if I have disagreements and conflicts with my students, my feelings of affection towards them do not change*).		0.673	
6. Mantengo miradas amables hacia ellos/as (*I maintain a friendly gaze towards them*).	0.433	0.611	
7. Transmito alegría en la relación con mi alumnado, y esto queda reflejado en mis expresiones (*I convey joy in my relationship with my students, and this is reflected in my expressions*).	0.370	0.539	
11. Estoy pendiente de su estado de ánimo para ayudarlos (*I am attentive to their mood in order to help them*).	0.447	0.491	
2. Me preocupo en buscar y aplicar nuevas metodologías de enseñanza (*I make an effort to find and apply new teaching methodologies*).			0.802
4. Realizo un seguimiento del aprendizaje de mi alumnado para evaluarlo mejor (*I monitor my students’ learning in order to evaluate them better*).			0.732
13. Soy exigente conmigo mismo/a para que mi alumnado aprenda lo mejor posible (*I am demanding of myself so that my students learn as well as possible*).		0.499	0.508

Note: PA (Loving proactivity); AC (Communicative affectivity), CD (Teaching compassion) and CI (Instructional commitment); Underlined item corresponds to AC.

**Table 10:** Internal consistency coefficients for each factor of the CUCODE, 14 item.

Components	Elements	α AFC	ω AFC
Global	14	0.98	0.98
Loving proactivity	4	0.97	0.98
Teacher compassion	3	0.93	0.95
Instructional commitment	3	0.94	0.95
Communicative affectivity	4	0.95	0.96

**Table 11:** Discrimination and difficulty parameters of items according to the response model.

	a1	d1	d2	d3	d4	d5	d6	d7
Item 1	3.350	10.443	7.658	4.064	2.550	1.550	0.280	−1.168
Item 2	3.467	11.619	8.156	4.932	3.430	2.267	0.910	−1.177
Item 3	4.045	13.175	10.022	5.821	3.880	2.832	1.299	−1.034
Item 4	3.663	11.400	8.879	5.182	3.569	2.610	1.020	−0.818
Item 5	6.316	20.153	16.389	9.535	6.294	4.284	2.145	−0.924
Item 6	5.970	18.557	14.663	8.308	5.271	3.644	1.713	−1.086
Item 7	5.564	16.869	13.439	7.773	4.626	3.095	1.184	−1.592
Item 8	6.976	21.523	16.394	10.003	5.994	4.146	2.037	−1.214
Item 9	6.173	17.969	15.894	9.145	5.707	4.157	2.098	−0.720
Item 10	4.860	14.466	12.304	6.888	4.703	3.297	1.611	−0.636
Item 11	4.039	12.780	10.128	6.229	4.310	3.302	1.460	−0.349
Item 12	6.405	18.755	14.911	9.048	6.155	4.294	1.811	−1.213
Item 13	4.330	12.940	9.610	5.589	3.470	1.651	0.270	−1.671
Item 14	1.519	3.353	2.310	1.398	0.771	0.185	−0.590	−1.495

**Table 12:** Correlations between CUCODE dimensions and the IRI, CCE, and TMMS-24 scales.

	PA	AC	CD	CI
IRI Perspective Taking	0.21 ***	0.22 ***	0.23 ***	0.22 ***
IRI Fantasy	0.067 **	0.034	0.026	0.023
IRI Empathic Concern	0.45 ***	0.50 ***	0.47 ***	0.50 ***
IRI Personal Distress	−0.08 ***	−0.11 ***	−0.12 ***	−0.11 ***
CCE Proactivity	0.24 ***	0.19 ***	0.18	0.15 ***
CCE Openness	0.24 ***	0.20 ***	0.20 ***	0.17 ***
CCE Listening	0.16 ***	0.15 ***	0.14 ***	0.13 ***
TMMS Awareness	0.12 ***	0.04 ***	0.03	0.02
TMMS Clarity	0.27 ***	0.21 ***	0.23 ***	0.17 ***
TMMS Repair	0.22 ***	0.16 ***	0.17 ***	0.13 ***

Note: PA (Loving Proactivity); AC (Communicative Affectivity), CD (Teaching Compassion) and CI (Instructional Commitment); IRI (Interpersonal Reactivity Index), CCE (Emotional Communication Questionnaire) and TMMS (Trait Meta Mood Scale); ** (*p* < .001); *** (*p* < .001).

## Data Availability

The data presented in this study are available on request from the author.

## Abbreviations

The following abbreviations are used in this manuscript:

AC	Affective Communication
BIC	Bayesian Information Criterion
CFA	Confirmatory Factor Analysis
CCE	Emotional Communication Questionnaire
CD	Teacher Compassion
CED	Teacher Emotional Commitment
CFI	Comparative Fit Index
CI	Instructional Commitment
CUCODE	Teacher Emotional Commitment Questionnaire
EFA	Exploratory Factor Analysis
IRI	Interpersonal Reactivity Index
IRT	Item Response Theory
KMO	Kaiser–Meyer–Olkin Test
PA	Loving Proactivity
RMSEA	Root Mean Square Error of Approximation
SRMR	Standardized Root Mean Square Residual
TLI	Tucker–Lewis Index
TMMS-24	Trait Meta-Mood Scale (24-item version)
WLSMV	Weighted Least Squares Mean and Variance adjusted

## Appendix A

**Cuestionario de Compromiso Emocional Docente (CUCODE)**

INSTRUCCIONES

A continuación, se presentarán una serie de sentencias que expresan posibles maneras de relacionarte como docente con tu alumnado.

La tarea que debes desarrollar es valorar, según la escala, *cuánto te identificas* con cada una de las sentencias, en función de que te veas reflejado/a en esas conductas.

Puedes estar de acuerdo con las frases, pero no tienes porqué identificarte totalmente con ellas, así que utiliza los diferentes grados de la escala para especificar el nivel de tu identificación con cada sentencia (1 = no me identifico nada, 8 = me identifico mucho). Marca tu elección para cada una de las sentencias en el rango establecido.

1. Las muestras de cariño son imprescindibles en mis clases.
2. Me preocupo en buscar y aplicar nuevas metodologías de enseñanza.
3. Realizo un seguimiento del aprendizaje de mi alumnado para evaluarlo mejor.
4. Aunque tenga roces y conflictos con mi alumnado, mi sentimiento de afecto hacia ellos/as no cambia.
5. Mantengo miradas amables hacia ellos/as.
6. Transmito alegría en la relación con mi alumnado, y esto queda reflejado en mis expresiones.
7. Cuando me dirijo hacia mi alumnado utilizo palabras de gratitud hacia ellos/as.
8. Soy generoso/a en la relación con mi alumnado: doy afecto sin esperar que ellos/as me lo devuelvan.
9. Estoy pendiente de su estado de ánimo para ayudarlos.
10. Soy capaz de perdonar a mi alumnado, aunque me haga daño.
11. Soy exigente conmigo mismo/a para que mi alumnado aprenda lo mejor posible.
12. Hablo con cariño cuando me relaciono con ellos.
13. Expreso a mi alumnado el amor que siento hacia ellos/as.
14. Suelo ser afectuoso/a con ellos/as a través de besos y/o abrazos

**Teacher Emotional Commitment Questionnaire (CUCODE)**

INSTRUCTIONS

Below you will find a series of statements describing possible ways of relating to your students as a teacher. 

Your task is to rate, according to the scale provided, the extent to which you identify with each statement, depending on how much you see yourself reflected in those behaviors.

You may agree with the sentences without fully identifying with them; therefore, use the different points on the scale to specify your level of identification with each statement (1 = I do not identify at all; 8 = I identify very much). Indicate your choice for each statement within the established range.

1. Showing affection is essential in my classes.
2. I make an effort to find and apply new teaching methodologies.
3. I monitor my students’ learning in order to evaluate them better.
4. Even if I have disagreements and conflicts with my students, my feelings of affection towards them do not change.
5. I maintain a friendly gaze towards them.
6. I convey joy in my relationship with my students, and this is reflected in my expressions.
7. When I address my students, I use words of gratitude towards them.
8. I am generous in my relationship with my students: I give affection without expecting them to return it.
9. I am attentive to their mood in order to help them.
10. I am able to forgive my students, even if they hurt me.
11. I am demanding of myself so that my students learn as well as possible.
12. I speak kindly when I interact with them.
13. I express my love for my students.
14. I tend to be affectionate with them through kisses and/or hugs.

Note: The items have been translated to facilitate understanding; however, it is essential to adapt the scale to the specific context in which it will be used.
